# Iatrogenic chylopneumopericardium in rheumatoid pericarditis

**DOI:** 10.1093/ehjcr/ytae543

**Published:** 2024-10-03

**Authors:** Dinesh P Raja, Sudipta Mondal, Arun Gopalakrishnan, Sivadasanpillai Harikrsihnan

**Affiliations:** Department of Cardiology, Sree Chitra Tirunal Institute for Medical Sciences and Technology, Thiruvananthapuram, Kerala 695011, India; Department of Cardiology, Sree Chitra Tirunal Institute for Medical Sciences and Technology, Thiruvananthapuram, Kerala 695011, India; Department of Cardiology, Sree Chitra Tirunal Institute for Medical Sciences and Technology, Thiruvananthapuram, Kerala 695011, India; Department of Cardiology, Sree Chitra Tirunal Institute for Medical Sciences and Technology, Thiruvananthapuram, Kerala 695011, India

## Case

**Figure 1:**
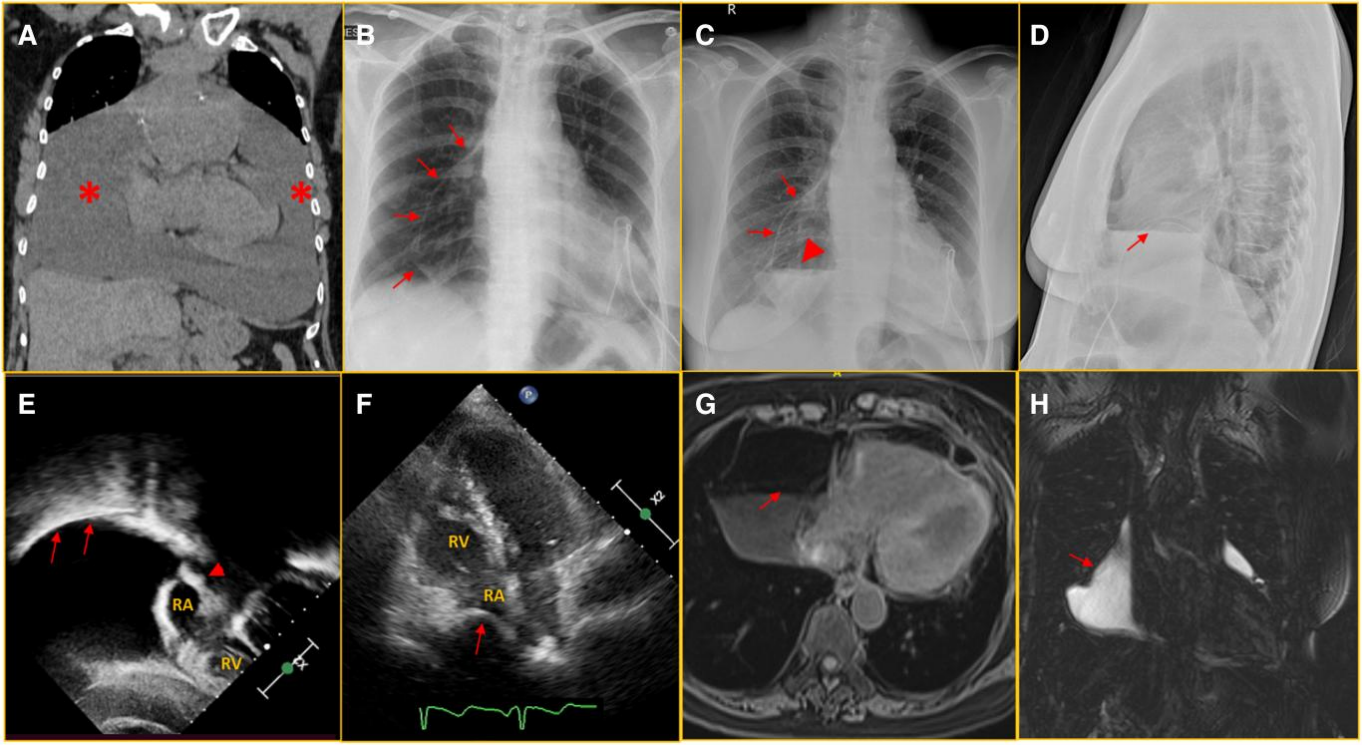
(*A*) Cardiac computed tomogram on coronal section showing massive pericardial effusion (asterisk—pericardial effusion). (*B*) Chest X-ray in anteroposterior view showing pneumopericardium (arrows—pericardium). (*C* and *D*) Chest X-ray in anteroposterior and lateral view (*D*) showing pneumopericardium (air–fluid level—arrowhead; arrows—pericardium). (*E*) 2D echocardiogram in modified subcostal four-chamber view showing pericardial effusion with air–fluid level (arrows) (arrowhead—interatrial septum). (*F*) 2D echocardiogram in apical four-chamber view showing pericardial effusion with diastolic right atrium collapse (arrow). (*G* and *H*) Cardiac magnetic resonance imaging in axial post-contrast T_1_ fat saturation (*G*) and coronal heavily T_2_-weighted (*H*) section confirming chylopneumopericardium (arrows—air–fluid level).

A 54-year-old female was diagnosed with massive pericardial effusion while being evaluated for constitutional symptoms with mild dyspnoea and a 4-month history of symmetric small joint pain and swelling with early morning stiffness suggesting inflammatory polyarthritis. A chest computed tomogram showed massive pericardial effusion (*Figure 1A*). In view of the haemodynamic compromise, a pigtail catheter was inserted and 1.5 L of serosanguinous fluid was drained. Initial pericardial fluid analysis revealed an exudative pattern. Her rheumatoid factor was 49.7 U/mL (normal <20 U/mL), and anti-cyclic citrullinated peptide was positive. Extensive infective and malignancy work-up were negative. Despite initial management, she had recurrent pericardial effusion for which she underwent surgical pericardial window creation and pericardial drain insertion. Post-operatively, pericardial effusion turned milky in colour, and biochemical analysis showed increased triglycerides (588 mg/dL) in the pericardial fluid, revealing it to be chyle. She was referred to the interventional cardiology team at this juncture for further management. A chest X-ray 1 day following the surgery revealed pneumopericardium (*Figure 1B*) subsequently found to be chylopneumopericardium with an air–fluid level in the pericardium (*Figure 1C* and *D*). Echocardiogram and cardiac magnetic resonance imaging confirmed the same (*Figure 1E–H*, see Supplementary material online, *Videos S1*–*S3*). Magnetic resonance lymphangiogram revealed a normal thoracic duct with no leak. She was continued on octreotide and disease-modifying anti-rheumatic drugs. The pericardial effusion changed serous, and the pericardial drain was removed after 48 h and she was discharged. A final diagnosis of rheumatoid arthritis associated with pericarditis with massive pericardial effusion with iatrogenic traumatic chylopneumopericardium was made.

Treatment for iatrogenic chylopneumopericardium primarily centres on preventing further chyle accumulation in the pericardium and mitigating the risk of tension pneumopericardium. If a discernible leak is identified on a lymphangiogram, either surgical or transcatheter closure of the thoracic duct or its tributaries is indicated. In the absence of a clear leak, conservative management is considered. To manage pneumopericardium, a conservative strategy with vigilant monitoring for tension pneumopericardium is often pursued. However, in cases where the risk is deemed high, a prophylactic pericardial window or pericardial drain may be placed.^1,2^ In instances of haemodynamic instability or tension pneumopericardium, immediate surgical decompression is imperative. In the presented case, a pigtail catheter was retained *in situ*, and a conservative approach was adopted.

## Supplementary Material

ytae543_Supplementary_Data

## Data Availability

All data are incorporated into the article and its online Supplementary material.

## Supplementary material

**Consent:** Obtained as per the COPE guidelines.

**Funding:** None declared.
